# AlloReverse: multiscale understanding among hierarchical allosteric regulations

**DOI:** 10.1093/nar/gkad279

**Published:** 2023-04-18

**Authors:** Jinyin Zha, Qian Li, Xinyi Liu, Weidong Lin, Tingting Wang, Jiacheng Wei, Ziliang Zhang, Xun Lu, Jing Wu, Duan Ni, Kun Song, Liang Zhang, Xuefeng Lu, Shaoyong Lu, Jian Zhang

**Affiliations:** State Key Laboratory of Functions and Applications of Medicinal Plants & School of Pharmacy, Guizhou Medical University, Guizhou550025, China; Wenzhou Medical University, Wenzhou325035, China; Medicinal Chemistry and Bioinformatics Center, Shanghai Jiao Tong University School of Medicine, Shanghai200025, China; Medicinal Chemistry and Bioinformatics Center, Shanghai Jiao Tong University School of Medicine, Shanghai200025, China; Medicinal Chemistry and Bioinformatics Center, Shanghai Jiao Tong University School of Medicine, Shanghai200025, China; Medicinal Chemistry and Bioinformatics Center, Shanghai Jiao Tong University School of Medicine, Shanghai200025, China; Nutshell Therapeutics, Shanghai201210, China; Medicinal Chemistry and Bioinformatics Center, Shanghai Jiao Tong University School of Medicine, Shanghai200025, China; Medicinal Chemistry and Bioinformatics Center, Shanghai Jiao Tong University School of Medicine, Shanghai200025, China; Wenzhou Medical University, Wenzhou325035, China; Medicinal Chemistry and Bioinformatics Center, Shanghai Jiao Tong University School of Medicine, Shanghai200025, China; Medicinal Chemistry and Bioinformatics Center, Shanghai Jiao Tong University School of Medicine, Shanghai200025, China; Department of Assisted Reproduction, Shanghai Ninth People’s Hospital, Shanghai Jiao Tong University School of Medicine, Shanghai 200011, China; Medicinal Chemistry and Bioinformatics Center, Shanghai Jiao Tong University School of Medicine, Shanghai200025, China; Wenzhou Medical University, Wenzhou325035, China; Nutshell Therapeutics, Shanghai201210, China; Department of Biomedical Sciences, College of Veterinary Medicine and Life Sciences, City University of Hong Kong, Hong Kong999077, China; Department of Assisted Reproduction, Shanghai Ninth People’s Hospital, Shanghai Jiao Tong University School of Medicine, Shanghai 200011, China; Medicinal Chemistry and Bioinformatics Center, Shanghai Jiao Tong University School of Medicine, Shanghai200025, China; Institute of Energy Metabolism and Health, Shanghai Tenth People’s Hospital, Tongji University School of Medicine, Shanghai200072, China; State Key Laboratory of Functions and Applications of Medicinal Plants & School of Pharmacy, Guizhou Medical University, Guizhou550025, China; Wenzhou Medical University, Wenzhou325035, China; Medicinal Chemistry and Bioinformatics Center, Shanghai Jiao Tong University School of Medicine, Shanghai200025, China; School of Pharmaceutical Sciences, Zhengzhou University, Zhengzhou450001, China

## Abstract

Increasing data in allostery are requiring analysis of coupling relationships among different allosteric sites on a single protein. Here, based on our previous efforts on reversed allosteric communication theory, we have developed AlloReverse, a web server for multiscale analysis of multiple allosteric regulations. AlloReverse integrates protein dynamics and machine learning to discover allosteric residues, allosteric sites and regulation pathways. Especially, AlloReverse could reveal hierarchical relationships between different pathways and couplings among allosteric sites, offering a whole map of allostery. The web server shows a good performance in re-emerging known allostery. Moreover, we applied AlloReverse to explore global allostery on CDC42 and SIRT3. AlloReverse predicted novel allosteric sites and allosteric residues in both systems, and the functionality of sites was validated experimentally. It also suggests a possible scheme for combined therapy or bivalent drugs on SIRT3. Taken together, AlloReverse is a novel workflow providing a complete regulation map and is believed to aid target identification, drug design and understanding of biological mechanisms. AlloReverse is freely available to all users at https://mdl.shsmu.edu.cn/AlloReverse/ or http://www.allostery.net/AlloReverse/.

## Graphical Abstract

**Graphical Abstract:**
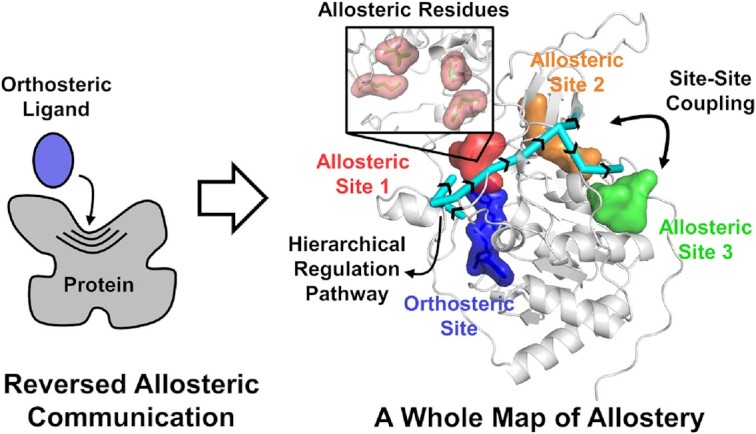
Identification of allosteric residues, allosteric sites, hierarchical regulation pathway and site-site couplings on protein surface.

## INTRODUCTION

Allostery (1–3) is the phenomenon that the function of an orthosteric site is regulated by a topologically distant allosteric site. Allostery is a fundamental way to tune the life process (4,5) and has been applied manually for the treatment of human diseases (6–9), namely designing drugs bound to allosteric sites to realize a ‘remote control’. Allosteric drugs are an alternative solution to classically undruggable therapeutic targets. They are better in safety and selectivity compared to orthosteric drugs (10–12).

Designing allosteric drugs requires multiscale knowledge of target proteins, including allosteric sites (13) for drug binding, allosteric residues for structure-based drug design and optimization, and residue pathways of allosteric regulation (14,15) for mechanism exploration. This information could be revealed experimentally by large-scale mutagenesis (16) but with a huge cost of time and resources. A more rational way is to computationally (17–19) predict allosteric positions before biological validation. Several *in silico* tools (20–23) such as AllositePro (24) for predicting allosteric sites and ProteinLens (25) for locating regulating pathways were established, which have helped rational and efficient design of allosteric modulators, for example the first activators for both sirtuin 6 (26) and glutathione peroxidase 4 (27). However, the accumulating data of allostery (28) pose new challenges. Many proteins have more than one allosteric site (29,30), and couplings among different allosteric sites have been observed theoretically (31) and experimentally (32–34). Currently, there are no easy-to-use tools to analyze biological relationships among different allosteric sites and regulations. Such relationships not only would show a complete map of allostery on protein surface, but also could hint at allosteric pharmacotherapy or design of bivalent drugs (35). Furthermore, there are limited tools for a complete multiscale analysis of protein allostery.

Here, we introduce AlloReverse, a web server for multiscale analysis of multiple allosteric regulations. AlloReverse is built based on our previously developed ‘reversed allosteric communication theory’ (36–39), suggesting that in addition to classical regulation, allosteric sites are also regulated by orthosteric sites (40). The reversed nature of the theory, which adopts one start point (orthosteric site) and multiple end points (allosteric sites), enables synchronous analysis of different allosteric regulations on proteins. AlloReverse adopts protein dynamics, machine learning (ML) and shortest pathway algorithm to discover allosteric residues, allosteric sites, hierarchical regulation pathways and couplings among predicted sites. Our model was benchmarked to discover known allosteric sites on 77.6% proteins. Importantly, we applied AlloReverse to explore global allostery on cell division cycle 42 GTP-binding protein (CDC42) and sirtuin 3 (SIRT3). Novel predicted allosteric sites on both proteins were validated experimentally, and a possible scheme for drug design was proposed. Collectively, AlloReverse is a novel workflow to boost understanding of allostery and allosteric drug design.

## MATERIALS AND METHODS

### AlloReverse server

**Figure 1.**
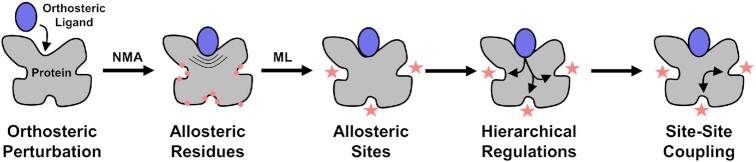
Workflow of AlloReverse.

AlloReverse is a server to predict multiscale allosteric information, including allosteric residues (residue scale), allosteric sites (domain scale) and allosteric pathways (protein scale), based on reversed allosteric communication theory. Especially, AlloReverse could reveal hierarchical relationships between different pathways and couplings among allosteric sites. The web server requires no login and is free to users around the world. The following parts are a brief overview of input, output and workflow (Figure 1). More details are given in Section S1 of Supplementary Data.

### AlloReverse input

The process of input contains three steps. First, a protein structure with orthosteric ligand bound is uploaded, either from RCSB Protein Data Bank (41) via a valid PDB ID or from a manual file in PDB format. Considering computational time, structure file >5 MB (roughly 6000 residues) is not allowed. Second, users are inquired whether to remove redundant chains. Finally, users could assign the orthosteric ligand in the structure file, either from a list of nonstandard residues or through manual specification.

### AlloReverse output

Results are shown in ‘Job Queue’ when the calculation is finished, which contains four parts, as shown in Supplementary Figure S1. The first part includes an interactive window displaying protein structure and a summary of job information. The second part is a list of predicted allosteric sites with their confidences. Residues making up each site could be demonstrated in display window by clicking the ‘Show Site’ button. Detailed information of each site would be shown in a pop-up window after clicking the ‘More Info’ button. The new window has a table of physicochemical properties of the specific site, a 2D display of residues by hydrophobicity and reversed allosteric effect (RAE), and a mapping of variants or mutations to this site by data recorded in UniProt (42). Residues with high RAEs are supposed to be allosteric residues in this potential site. The third part is a figure of hierarchical residue pathways. In the figure, the orthosteric site is represented by a red circle in the middle, the predicted allosteric sites are shown by orange circles and residues in the pathways are displayed by blue circles. Arrows linking circles represent the routes of reversed allosteric communication. This diagram offers users with a whole map of allostery on protein surface and coupling relationships among different regulations. The last part is a heatmap describing regulations among predicted allosteric sites. The values range from 0 (white) to 1 (blue), where a larger value suggests a stronger regulation. Strongly coupled allosteric sites might be used for combined pharmacotherapy and design of bivalent modulators.

All data could be downloaded by clicking the ‘Download Report’ button. Running time of AlloReverse ranges from 1 min for a protein with <400 amino acids to half an hour for a protein with roughly 6000 amino acids. A step-by-step tutorial could be found in the ‘Help’ page on the server.

### Identification of allosteric residues

Pocket-like regions are first located on protein surface geometrically (43). RAE of a residue is then defined as its change of residue–residue interactions in the pocket between the *apo* and orthosteric ligand-bound (*holo*) states (44). RAE suggests response of each residue against orthosteric perturbation. Residues with high RAE are supposed to be allosteric residues.

### Recognition of allosteric sites

Allosteric sites are recognized with an AdaBoost classifier (28,45–47), by judging which pocket-like regions detected in the previous step are allosteric sites. AdaBoost is a serial ensemble learning method, which trains the new classifier focusing on misclassified samples of the old classifiers. The classifier adopts hydrophobicity, flexibility and RAE of the pocket (sum of RAEs of residues in it) as input features. The model would also output prediction confidence for each potential site.

### Prediction of hierarchical regulation pathways

An important feature of AlloReverse is that it could predict all allosteric regulations in one shot and analyze their coupling relationships, based on the reverse nature of reversed allosteric communication theory. Following previous efforts on allosteric mechanisms (48–50), regulation pathway of a predicted site is defined to be the shortest route from an orthosteric ligand to the residue in site with the highest RAE, where ‘distances’ between residues are calculated as the reciprocal of their mean motion correlation.

### Evaluation of site–site coupling

Since different regulation pathways would share some residues, it is believed that these allosteric sites might influence each other. Based on a previous hierarchical regulation pathway, the degree of site A coupled by site B is defined as the proportion of shared residues in the pathway toward site A, i.e. the coupling is usually asymmetric. Strongly coupled sites may be applied for combined allosteric therapy or design of bivalent drugs.

## PERFORMANCE OF ALLOREVERSE

The performance of AlloReverse relies on how good the ML model could discriminate allosteric sites from pocket-like regions. The power of classification was benchmarked on a test set (Supplementary Figures S2–S5 and Supplementary Table S2) of 58 proteins, which contains 926 pocket-like regions and 83 of them are labeled as ‘allosteric sites’. The distribution of descriptor values and the ratio of labeled sites in the test set were found to be statistically same as those in the training set (Supplementary Figure S4 and Supplementary Table S1), ensuring the validity of benchmarking. Our model could recall 71.0% labeled allosteric sites and could re-emerge at least one allosteric site for 77.6% proteins in the test set. These data have shown good sensibility of the model in recognizing allosteric sites on complex protein surface. The classification power was further testified by receiver operating characteristic (ROC) curve (20) (Supplementary Figure S6) of the model. The area under ROC curve was calculated to be 0.758, so that AlloReverse could significantly differentiate allosteric sites from other sites. All these data have ensured the predicting power of AlloReverse.

## EXAMPLES

### Case 1: allosteric sites and allosteric residues on CDC42

**Figure 2.**
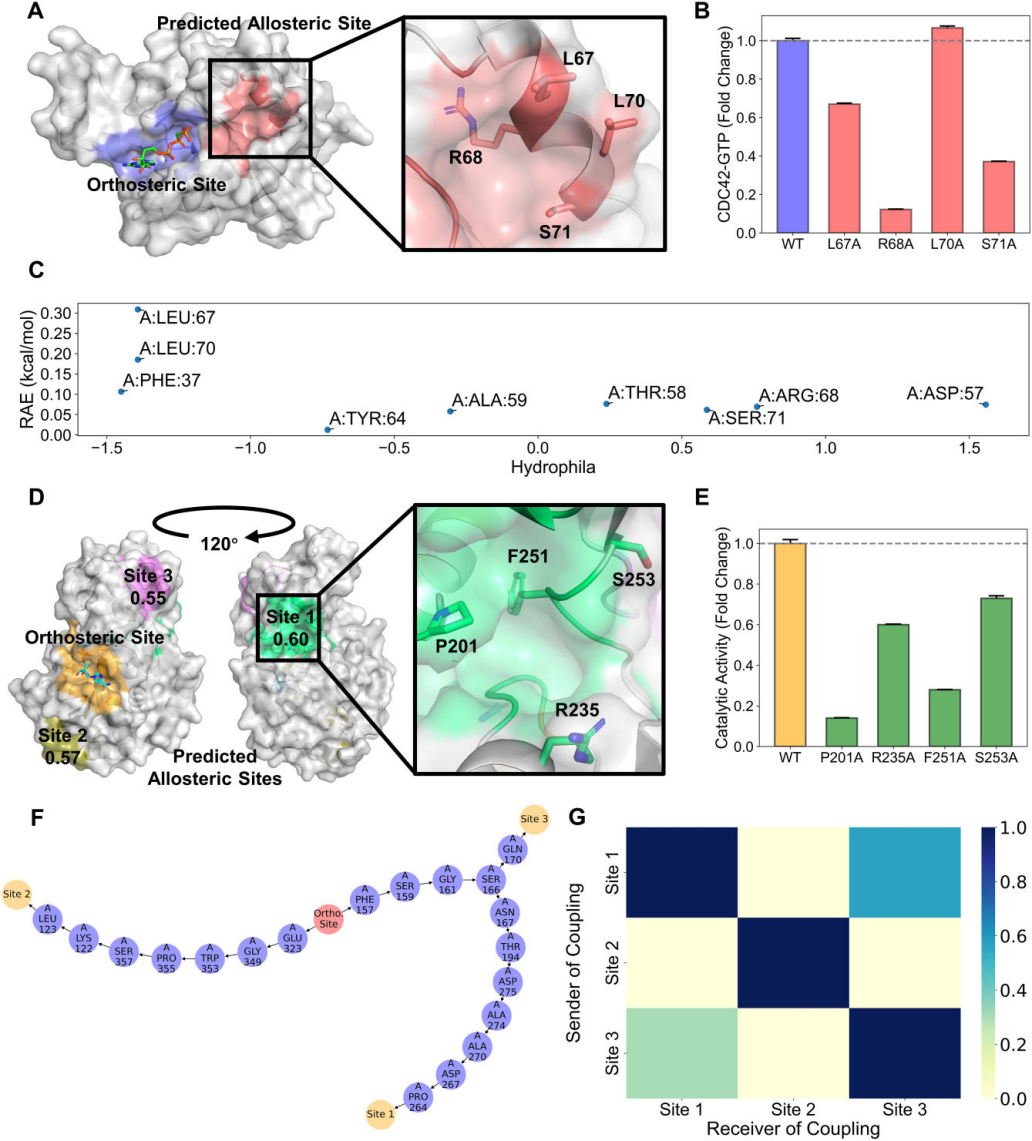
Examples of AlloReverse. CDC42: (**A**) Novel allosteric site predicted by AlloReverse. (**B**) Validation of allosteric site by mutagenesis. The *Y*-axis means the degree of GTP binding by CDC42. (**C**) RAE of residues in the novel allosteric pocket. SIRT3: (**D**) Novel allosteric sites predicted by AlloReverse. Value under site name represents the prediction confidence. (**E**) Validation of allosteric site by mutagenesis. The *Y*-axis means the degree of deacetylation by SIRT3. (**F**) Hierarchical regulation pathway predicted on SIRT3. (**G**) Site–site couplings predicted on SIRT3.

CDC42 is a GTPase regulating cytoskeleton during cell division (51,52). It is a star target in the field of immunosuppression and anti-inflammation (53). There are no reported allosteric sites on CDC42. We analyzed CDC42 with GMP bound at the orthosteric site [PDB ID 2QRZ (54)] using AlloReverse. An allosteric site close to the GMP orthosteric site was predicted, as shown in Figure 2A, formed by an α-helix (residues 65–70) and a loop (residues 56–64). To validate the prediction, we performed site-directed mutagenesis, including L67A, R68A, L70A and S71A. The four residues, which are on the α-helix, were chosen because they are relatively distant from the orthosteric site. We found that the decreased degree in the GTP binding levels was significantly observed in the L67A, R68A and S71A variants (Figure 2B). CDC42 is activated by GTP binding. Thus, the decreased GTP binding levels in the variants suggest the potential of this site in the regulation of CDC42 activity (see Section S2 of Supplementary Data andSupplementary Figure S7A for experimental details). Interestingly, we found that L67 was also predicted to be an allosteric residue (Figure 2C), with the highest RAE in the site. These data have demonstrated the power of discovering a novel allosteric site and key residues using AlloReverse.

### Case 2: allosteric sites and hierarchical regulating pathways on SIRT3

SIRT3 is a deacetylase able to regulate many proteins in mitochondria (55,56). It plays important roles in development of cancer and cardiovascular disease (57,58). Previous research works have reported two shallow allosteric sites on SIRT3 [PDB IDs 4C78 (59) and 5Y4H (60)]. We analyzed SIRT3 with carba-NAD bound at the orthosteric site [PDB ID 4FVT (54)] using AlloReverse. As shown in Figure 2D, three allosteric sites were predicted. All three sites are located differently to the previous report, although site 2 is close to the ligand of 4C78 (Supplementary Figure S8). The validity of site 1, which has the highest confidence, was further proved by mutagenesis (see Section S2 of Supplementary Data andSupplementary Figure S7B for experimental details), where a significant decrease in deacetylation activity was found in response to the single P201A, R235A, F251A and S253A variants in the site (Figure 2E). In addition, sites 1 and 3 are coupled since they share four residues on regulation pathways, while regulation toward site 2 adopts a completely different direction (Figure 2F and G). This result suggests that further study could focus on both sites 1 and 3 for design of bivalent molecules or allosteric pharmacotherapy.

## DISCUSSION

Allostery is a remarkable technique for drug design, especially for classically undruggable targets (6). Allosteric drugs are praised for better safety, selectivity and functional diversity (7,8). Though many computational tools have already been developed to facilitate difficulties in studying allostery (22), accumulating data (28) are calling for coupling analysis among different allosteric regulations on a single protein, which might hint at the design of bivalent drugs and combined allosteric drug use. Also, tools are required to produce different scales of allosteric data, including allosteric residues (residue scale), allosteric sites (domain scale) and allosteric pathways (protein scale) in one shot. Under such demand, we have introduced AlloReverse, a web server for analyzing multiscale and multiple allosteric regulations on protein surface. The server could predict regulating residues, allosteric sites, allosteric pathways and site–site couplings based on reversed allosteric communication theory. Importantly, AlloReverse could reveal the hierarchical relationships among different regulations. AlloReverse was applied for predicting novel allosteric sites and site–site couplings on CDC42 and SIRT3. Currently, AlloReverse requires orthosteric ligand-bound structure as input, which is numbered. Further effort could be focused on automatic assignment of orthosteric perturbations in the *apo* structures. In addition, the prediction of allosteric sites and site–site couplings requires further validation. Cumulatively, AlloReverse is a novel workflow for discovering a whole map of allostery. It is believed to accelerate the design of allosteric drugs.

## DATA AVAILABILITY

AlloReverse is freely available to all users at https://mdl.shsmu.edu.cn/AlloReverse/ or http://www.allostery.net/AlloReverse/.

## Supplementary Material

gkad279_Supplemental_File
