# Supplementary material for: AlloReverse: multiscale understanding among hierarchical allosteric regulations
Source: Nucleic Acids Res. 2023 Apr 18;51(W1):W33–8. doi: 10.1093/nar/gkad279 (PMC10320067; doi:10.1093/nar/gkad279)
Supplement: gkad279_Supplemental_File [file gkad279_supplemental_file.pdf]

AlloReverse: Multiscale Understanding Among Hierarchical Allosteric Regulations

Supplementary Information

Jinyin Zha[1,2,3,†], Qian Li[3,†], Xinyi Liu[3,†], Weidong Lin[3,4,†], Tingting Wang[3,†], Jiacheng Wei[3], Ziliang Zhang[2], Xun Lu[3], Jing Wu[3,6], Duan Ni[3], Kun Song[2,4], Liang Zhang[5], Xuefeng Lu[6,*], Shaoyong Lu[3,7,*], Jian Zhang[1,2,3,8,*]

[1] State Key Laboratory of Functions and Applications of Medicinal Plants & School of Pharmacy, Guizhou Medical University, Guizhou, 550025, China.
[2] Wenzhou Medical University, Wenzhou, 325035, China.
[3] Medicinal Chemistry and Bioinformatics Center, Shanghai Jiao Tong University School of Medicine, Shanghai, 200025, China.
[4] Nutshell Therapeutics, Shanghai, 201210, China.
[5] Department of Biomedical Sciences, College of Veterinary Medicine and Life Sciences, City University of Hong Kong, Hong Kong, 999077, China.
[6] Department of Assisted Reproduction, Shanghai Ninth People's Hospital, Shanghai Jiao Tong University School of Medicine, Shanghai, 200011, China.
[7] Institute of Energy Metabolism and Health, Shanghai Tenth People's Hospital, Tongji University School of Medicine, Shanghai 200072, China.
[8] School of Pharmaceutical Sciences, Zhengzhou University, Zhengzhou, 450001, China.

Corresponding Author
Jian Zhang
Email: Jian.zhang@sjtu.edu.cn
Tel: +86-21-63846590
Fax: +86-21-64154900
Shaoyong Lu
Email: lushaoyong@sjtu.edu.cn
Tel: +86-21-63846590
Fax: +86-21-64154900
Xuefeng Lu
Email: xuefenglu163@163.com
Tel: +86-21-63846590
Fax: +86-21-64154900

† The authors wish it to be known that, in their opinion, the first 5 authors should be regarded as joint First Authors.

Contents

Section S1: Detailed Introduction of AlloReverse. 3
1, Identification of Allosteric Residues with Reversed Allosteric Effect (RAE). 3
2, Recognition of Allosteric Sites. 3
3, Prediction of Hierarchical Regulation Pathways. 4
4, Evaluation of Site-Site Couplings. 5
Section S2: Experimental Details of Biological Validations. 6
1, Allosteric Site and Allosteric Residues on CDC42. 6
2, Allosteric Site and Hierarchical Regulating Pathways on SIRT3. 6
Section S3: Figures. 8
Section S4: Tables. 16

Section S1: Detailed Introduction of AlloReverse.

1, Identification of Allosteric Residues with Reversed Allosteric Effect (RAE).

Reversed allosteric effect (RAE) reflects response of a single residue against orthosteric perturbation. Those with strong RAE are identified as allosteric residues. RAE is mathematically defined as the change of residue-residue interactions inside a pocket between *apo* state and orthosteric ligand bound (*holo*) state, and was previously calculated using MM/GBSA decomposition along conformation ensemble generated by all-atom MD simulation (1,2). The main effort here, is to simplify the calculation.

Firstly, the input protein structure (binding orthosteric ligand) is reduced to coarse-grained (CG) models for the two states. In *apo* state, CG sites are placed at C_αs of the protein. In *holo* state, atoms of orthosteric ligand are also turned into CG sites. For both systems, the potential energy function (3) is:

$$E = \sum_{i=0}^{n} \sum_{j=i+1}^{n} \frac{1}{2} \cdot k_{ij} \cdot \left(d_{ij} - d_{ij}^0\right)^2 \cdot H\left(r_c - d_{ij}^0\right) \quad (1)$$

n is the number of CG sites. d_{ij} is distance between site i and j. d_{ij}^0 is the initial distance between site i and j. $H(x)$ is a Heaviside function. It equals 0 when $x \le 0$ and 1 when $x > 0$. k_{ij} is elastic constant between site i and j and is set as 1 kcal/(mol·Å). The cutoff distance r_c is set as 12 Å.

Then we can calculate the interaction between residue i to all other residues in candidate pocket P (detected by FPocket (4)) according to eqn. (1):

$$I_i^s = \sum_{\substack{j \in P \\ j \neq i}} \frac{1}{4} \cdot k_{ij} \cdot \left(\langle d_{ij} \rangle^s - d_{ij}^0\right)^2 \quad (2)$$

An additional one-second is multiplied here to avoid repeated calculation. Heaviside function is omitted here since every two residues in the pocket could have interactions. $\langle d_{ij} \rangle^s$ is the average distance between site i and j. s defines the state and is either *apo* or *holo*. $\langle d_{ij} \rangle^s$ is calculated by NMA. In brief, eigenvectors with corresponding eigenvalues are solved from the Hessian matrix of eqn. (1). These data describe the oscillations (also known as normal modes) from initial position with their frequencies for each CG site (or residue). Suppose for residue i, its displacement due to any of the normal modes obeys a normal distribution, we could describe the position ensemble of this residue at state s by:

$$R_i^s = R_i^0 + sf \sum_{p=1}^{q} X_p \cdot \lambda_p^{-0.5} \cdot u_p \quad (3)$$

, where R_i^0 describes the initial position of residue i, q is the number of modes used and is set to 100 here. λ_p and u_p are the eigenvalues and eigenvectors of mode p. sf is a scaling factor, ensuring the root mean square distance of the whole protein is 1 Å. X_p is a random variable obeying normal distribution. Therefore, $\langle d_{ij} \rangle^s$ is written as:

$$\langle d_{ij} \rangle^s = \langle \left| R_i^s - R_j^s \right| \rangle \quad (4)$$

Unluckily, this expectation could not be analytically solved. Therefore, we solved this approach with a Monto-Carlo approach with 2000 samples. We found, though final results are not identical in 50 runs, the deviations are negligible, and it will not change the final prediction of allosteric sites.

Finally, RAE of residue i could be written as:

$$RAE_i = I_i^{holo} - I_i^{apo} \quad (5)$$

Normal mode analysis and pose sampling is done by Prody (5) package.

2, Recognition of Allosteric Sites.

2.1, Model Construction.

AlloReverse identifies allosteric sites by discriminating whether a pocket-like region is allosteric or not using AdaBoost, a machine learning (ML) technique. AdaBoost is an ensemble learning framework using a serial of base models (6). The ith model is trained to patch the mistakes by the $(i-1)^{th}$ model. The model applies hydrophobicity, flexibility and RAE of pocket as input, and outputs judgement with prediction confidence. All three features are standardized according to protein. One-layer decision tree is applied as

the base model. The model was trained on a dataset of 134 proteins and tested on a dataset of 58 proteins. In training set, 2431 pocket like regions are detected, and 208 of which with at least 10% overlap of recorded allosteric ligand are labeled as "allosteric site", while the rest are labeled as "other site". Due to great imbalance (about 1:10) between the two sites, oversampling method SMOTE was applied in training. Best super-parameters, including number of classifiers and learning rate, were grid-searched based on 5-fold cross validation on training set, where the average Matthews correlation coefficient (MCC) was used for index. As shown in Figure S4, the best combination is 12 classifiers with a learning rate of 0.5, with an averaged MCC of 0.242. The final model, on one hand, could recall 75.0% known allosteric sites in the training set. On the other hand, it could re-emerge at least one allosteric site for 82.8% proteins in training set. The area under RUC curve is found to be 0.786. The model is constructed by imblearn and scikit-learn toolkit (7).

2.2, Dataset Collection.

A dataset of 192 proteins was firstly constructed, based on ASBench (8) and our manual collection (9). The proteins by manual collections were selected with the same criteria as ASBench. These proteins all bound to an orthosteric ligand. FPocket was then used to detect pockets on the 192 systems, and in consequence 3357 pockets were found. For labeling these pockets, allosteric ligands were aligned to these proteins, using allosteric ligand binding structure. A pocket was labeled "allosteric site" if it has at least a 10% overlap with an aligned allosteric ligand, otherwise it is "other site". After that, 291 pockets were labeled as "allosteric site", as shown in Figure S1. Note that this doesn't mean that there are 291 allosteric sites on the 192 proteins, because FPocket could split a real allosteric site into 2 or more sub-pockets (Figure S3). Since sub-pockets could be discrepant in allosteric response, it is acceptable if AlloReverse only recognize some of them. Such redundancy is removed while calculating the recall score of the model. Finally, the dataset was split into a training set of 134 proteins and test set of 58 proteins. The balance of distribution of feature values and ratio of different labels is proved in Figure S4, ensuring the validity of model benchmarking. All data are listed in Table S1 and S2.

2.3, Calculation of Input Features.

Hydrophobicity of pocket. This term is calculated with Fpocket. We would introduce how Fpocket detect pockets first. Fpocket first assigns alpha spheres in protein, which is a sphere decided by any 4 atoms in protein but with a restriction of radius range. Each alpha sphere decides a minor hole on protein. These holes are then clustered into several pockets on protein surface. In Fpocket, if more than 2 atoms, for decision of an alpha sphere, are polar atoms, then this alpha sphere is a polar alpha sphere. In Pocket P, we could calculate, for each polar alpha sphere, the number of other alpha spheres having overlap with current alpha sphere (n_{neigh}). Then hydrophobicity of pocket P, or "Mean local hydrophobic density" in Fpocket, is calculated as the average of n_{neigh} in pocket P.

Flexibility of pocket. This term is also calculated with Fpocket. Fpocket would define the atoms to form the pocket. The average B-factor among these atoms is then calculated. The value is finally normalized among all detected pockets on the protein surface.

RAE of pocket. RAE of the pocket is defined as the sum of RAE of residues in the pocket.

2.4, Empirical Adjustment After Prediction.

Prediction confidence is an output in AlloReverse by function "predict_proba" in scikit-learn toolkit. However, this value is not calibrated. We calibrate this value with the following relationship.

$$conf^{\Theta} = \min(2.5 * conf - 0.75, 1) \quad (6)$$

Due to oversampling applied, the model tends to give more positive predictions than reality. Therefore, we did the following adjustments. Pockets having more than 10% overlap with the orthosteric ligand is not a predicted allosteric site. In cases where above 70% pockets on protein are predicted to be allosteric sites, only sites in top 3 predicting confidence are selected as final prediction.

3, Prediction of Hierarchical Regulation Pathways.

Suppose the *holo* CG model is a graph and CG sites serve as nodes, regulation pathway toward pocket P is defined as the shortest route from the most central node of orthosteric ligand to the residue in pocket P with the highest RAE. The distance between every 2 nodes is defined as followed:

$$D_{ij} = \begin{cases} \frac{1}{corr_{ij}} & d_{ij}^0 \le 8\ \text{Å} \\ +\infty & d_{ij}^0 > 8\ \text{Å} \end{cases} \quad (10)$$

Here, d_{ij}^0 is the initial distance between site *i* and *j*; $corr_{ij}$ is the mean of Pearson correlation coefficient between node i and j at any direction or Top 100 modes. The shortest route is solved by Dijkstra algorithm (10).

4, Evaluation of Site-Site Couplings.

If pathway towards pocket P and Q each makes a set of residues named W_P and W_Q, then the site-site coupling score of pocket P by pocket Q is defined as:

$$c_{Q\to P} = \frac{\|W_P \cap W_Q\|}{\|W_P\|} \quad (11)$$

namely, the ratio of residues of pathway towards P shared by pathway towards Q. It could be seen that site-site coupling is asymmetry.

Reference

1. Zhang, Q., Chen, Y., Ni, D., Huang, Z., Wei, J., Feng, L., Su, J.-C., Wei, Y., Ning, S., Yang, X. *et al.* (2022) Targeting a cryptic allosteric site of SIRT6 with small-molecule inhibitors that inhibit the migration of pancreatic cancer cells. *Acta. Pharm. Sin. B*, **12**, 876-889.
2. Ni, D., Wei, J., He, X., Rehman, A.U., Li, X., Qiu, Y., Pu, J., Lu, S. and Zhang, J. (2020) Discovery of cryptic allosteric sites using reversed allosteric communication by a combined computational and experimental strategy. *Chem. Sci.*, **12**, 464-476.
3. Bahar, I. and Rader, A.J. (2005) Coarse-grained normal mode analysis in structural biology. *Curr. Opin. Struct. Biol.*, **15**, 586-592.
4. Le Guilloux, V., Schmidtke, P. and Tuffery, P. (2009) Fpocket: An open source platform for ligand pocket detection. *BMC Bioinformatics*, **10**, 168.
5. Zhang, S., Krieger, J.M., Zhang, Y., Kaya, C., Kaynak, B., Mikulska-Ruminska, K., Doruker, P., Li, H.C. and Bahar, I. (2021) ProDy 2.0: increased scale and scope after 10 years of protein dynamics modelling with Python. *Bioinformatics*, **37**, 3657-3659.
6. Freund, Y. and Schapire, R.E. (1997) A decision-theoretic generalization of on-line learning and an application to boosting. *J. Comput. Syst. Sci.*, **55**, 119-139.
7. Pedregosa, F., Varoquaux, G., Gramfort, A., Michel, V., Thirion, B., Grisel, O., Blondel, M., Prettenhofer, P., Weiss, R., Dubourg, V. *et al.* (2011) Scikit-learn: Machine Learning in Python. *J. Mach. Learn. Res.*, **12**, 2825-2830.
8. Huang, W., Wang, G., Shen, Q., Liu, X., Lu, S., Geng, L., Huang, Z. and Zhang, J. (2015) ASBench: benchmarking sets for allosteric discovery. *Bioinformatics*, **31**, 2598-2600.
9. Liu, X., Lu, S., Song, K., Shen, Q., Ni, D., Li, Q., He, X., Zhang, H., Wang, Q., Chen, Y. *et al.* (2020) Unraveling allosteric landscapes of allosterome with ASD. *Nucleic Acids Res.*, **48**, 394-401.
10. Abdelghany, H.M., Zaki, F.W. and Ashour, M.M. (2022) Modified Dijkstra Shortest Path Algorithm for SD Networks. *Int. J. Electr. Comput.*, **13**, 203-208.

Section S2: Experimental Details of Biological Validations.

1, Allosteric Site and Allosteric Residues on CDC42.

1.1, Expression and Purification of Wild-Type and Mutant CDC42.

The wild-type CDC42 gene was synthesized and cloned into a vector pET-32a (+) by Sangon Biotech (Shanghai) Co., Ltd. Site-directed mutagenesis (L67A, R68A, L70A and S71A) were introduced into the plasmid encoding the wild-type CDC42 by Mut Express II Fast Mutagenesis Kit V2 (Vazyme Biotech co.,ltd, Nanjing, Jiangsu, China). The successful constructions of plasmids were verified by DNA sequencing (Personalbio, Shanghai, China). The recombinant proteins were produced in Escherichia coli BL21 (DE3) (Weidi, Shanghai, China) and the expression was induced by 0.3 mM isopropyl β-D-1-thiogalactopyranoside (IPTG) (Sigma-Aldrich, St. Louis, USA) at 15°C.

The precipitated bacterium was lysed with a high-pressure French Press homogenizer AH-1500 (ATS Engineering, China) at 800 bar, 5 min and 4°C; the supernatant flowed through the Hi-Trap FF Ni^{2+} chelating column (GE Healthcare, USA) and Superdex 75 Gel Filtration Columns (GE Healthcare, USA) by ÄKTA EXPLORER (GE Healthcare, USA). The purified protein was examined by SDS-PAGE electrophoresis.

1.2, Determining the Degree of Activation of CDC42.

Referring to the previous detection method of CDC42 activity based on a high-throughput flow cytometry instrument (1), we established a simple detection method of cdc42 activity based on microplate reader. To measure the degree of GTP binding in CDC42, by which would activate CDC42, we mixed 3 µM wild-type or mutant CDC42 with 100 nM BODIPY-FL-GTP (Thermo G12411, USA) and 60 uL Ni^{2+}-Sepharose excel resin (Cytiva 17371202, USA) at total volume 300 µL complemented with NP-HPSA buffer [(0.01% (vol/vol) NP-40, 30 mM HEPES pH 7.5, 100 mM KCl, 20 mM NaCl, 1 mM EDTA, 0.1% BSA and 1 mM DTT) after the Ni^{2+}-Sepharose was washed twice by 300 µL ice-cold NP-HPSA buffer. This reaction was conducted at 37°C with shaking at 180 rpm for 45 min. Then, the mix was centrifuged at 3000 rpm for 3 min (Thermo, USA) and the precipitate was washed twice by NP-HPSA buffer and collected at 4°C Next, 300 µl NP-HPSA buffer supplemented with 500 mM imidazole, was mixed with the precipitate, and then the supernatant was collected into a microplate (Corning 3915, USA) after being centrifuged at 3000 rpm for 3 min (Thermo, USA). Light scatter and fluorescence emission at 485 nm and 530 nm from the supernatant were monitored on a multimode microplate reader Synergy Neo (Biotek, Winooski, USA). The level of active-form mutant CDC42 was normalized by wild-type CDC42.

2, Allosteric Site and Hierarchical Regulating Pathways on SIRT3.

2.1, Expression and Purification of Wild-Type and Mutant SIRT3.

The full-length human SIRT3 expression plasmid was a gift from Prof. Jinke Cheng at the Shanghai Jiao Tong University. Truncated SIRT3 (residues 118-399) was cloned into pET-28a (+) vector using the NdeI and BamHI restriction sites adding an N-terminal 6× histidine tag to ease protein purification. Site-directed mutagenesis (P201A, R235A, F251A and S253A) were introduced into the plasmid encoding the wild-type SIRT3-(118-399) by Mut Express II Fast Mutagenesis Kit V2 (Vazyme Biotech co.,ltd, Nanjing, Jiangsu, China). The successful constructions of plasmids were verified by DNA sequencing (Personalbio, Shanghai, China).

Plasmid was transformed into *E. coli* BL21 (DE3) cells (Weidi, Shanghai, China) for protein purification. A single colony was inoculated in 10 mL LB media containing 50 µg/ml kanamycin at 37 °C, 250 rpm overnight. The culture was then transferred to 1 L LB media until the A600 reached 0.6-0.8. The SIRT3 protein expression was induced with 0.3 mM isopropyl β-D-1-thiogalactopyranoside (IPTG) (Sigma-Aldrich, St. Louis, USA) at 16°C, 180 rpm overnight. Cells were collected by centrifugation, and the pellet was resuspended in lysis buffer (25 mM HEPES, pH 7.5, 250 mM NaCl, 5% glycerol) with fresh protein inhibitor cocktail (ApexBio, Houston, USA). Cells were lysed by French Press at 800 bar, 5 min and 6°C. Supernatant was separated from cell debris by centrifugation at 23,000 g for 1 h at 10°C and loaded onto a Ni-NTA column (Qiagen, Tehran, IRAN) that equilibrated with the lysis buffer. The column was washed with 10 column volumes of the lysis buffer additional containing 20 mM imidazole, and 5 column volumes of the lysis buffer additional containing 40 mM imidazole. Then the protein was eluted with the lysis buffer

additional containing 250 mM imidazole. The protein was further purified by HiTrap Q HP column (GE healthcare, Little Chalfont, UK) to 95% purity as assessed by SDS-PAGE analysis stained by Coomassie Brilliant Blue (One-step blue protein gel strain, Biotuim, San Francisco, USA). Finally, SIRT3 wild-type and mutant proteins were exchanged to SIRT assay buffer (50 mM Tris-HCl pH 8.0, 137 mM NaCl, 2.7 mM KCl, 1 mM $MgCl_2$) and stored at -80°C after quick freezing with liquid nitrogen.

2.2, Determining the Catalytic Activity of SIRT3.

To measure the activity of deacetylation by SIRT3, a 50 μL reaction system contained 75 μM Ac-RHKK(Ac)-AMC (substrate), 2.5 mM NAD^+, 1.25 μM SIRT3 and SIRT assay buffer. The deacetylation reactions were conducted at room temperature for 45 min, terminated with 40 mM nicotinamide, and developed with 6 mg/mL trypsin for 30 min. Fluorescence was monitored by a multimode microplate reader Synergy Neo (Biotek, Winooski, USA) with excitation at λ = 360 nm and emission at λ = 460 nm. The deacetylase activities of SIRT3 mutant were normalized by SIRT3 wild-type.

Ac-RHKK(Ac)-AMC used was synthesized and purchased from GL Biochem (Shanghai, China). The peptide was purified by HPLC to > 98% purity. The chemical structure was validated by MS analyses. NAD^+ (V900401), trypsin (T4799) and nicotinamide (N0636) were purchased from Sigma-Aldrich (St. Louis, USA).

Reference

1. Surviladze, Z., Waller, A., Wu, Y., Romero, E., Edwards, B.S., Wandinger-Ness, A. and Sklar, L.A. (2010) Identification of a Small GTPase Inhibitor Using a High-Throughput Flow Cytometry Bead-Based Multiplex Assay. *J Biomol Screen*, **15**, 10-20.

Section S3: Figures.

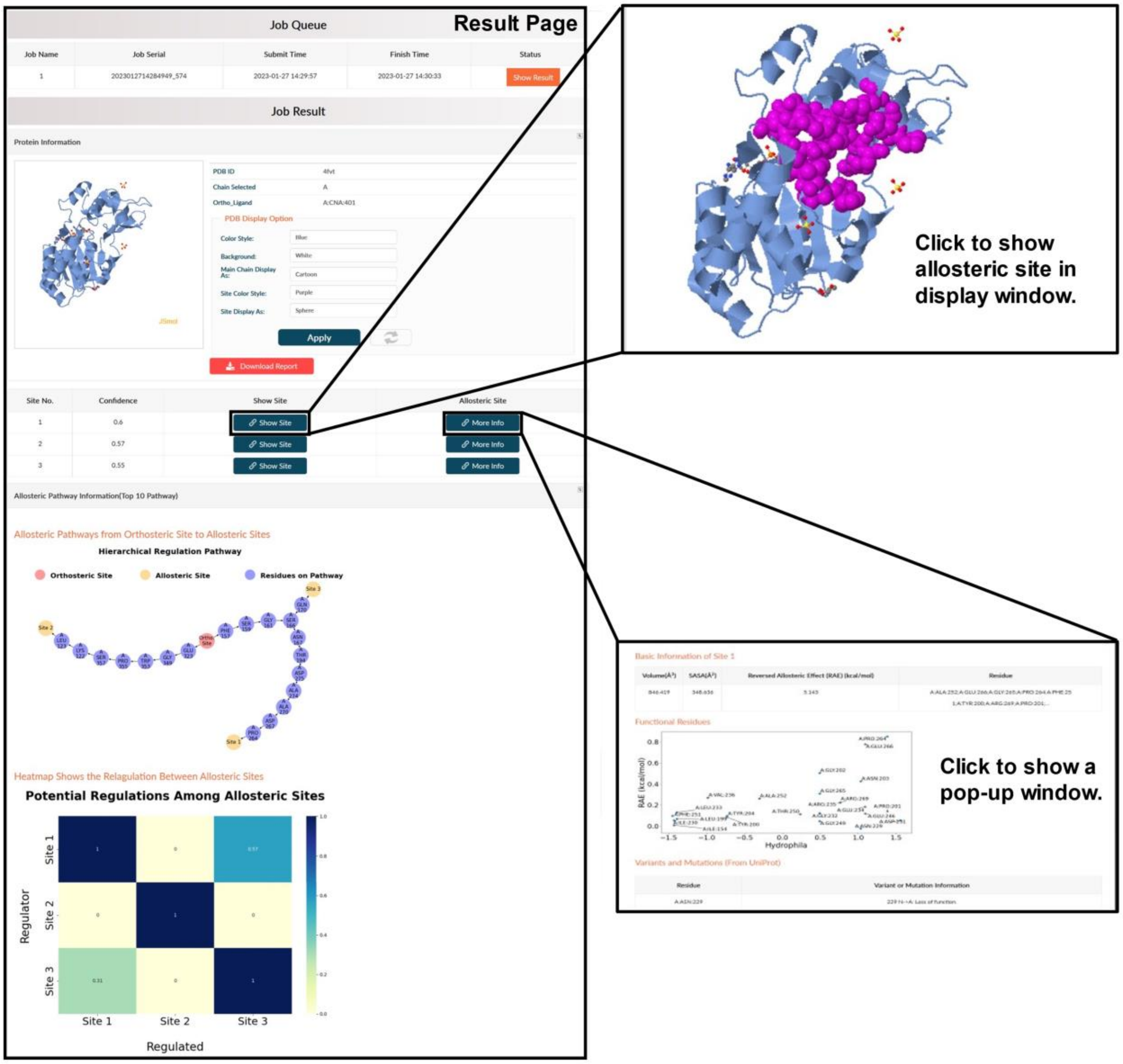

Figure S1. Result page of AlloReverse analysis of SIRT3 (PDB: 4FVT).

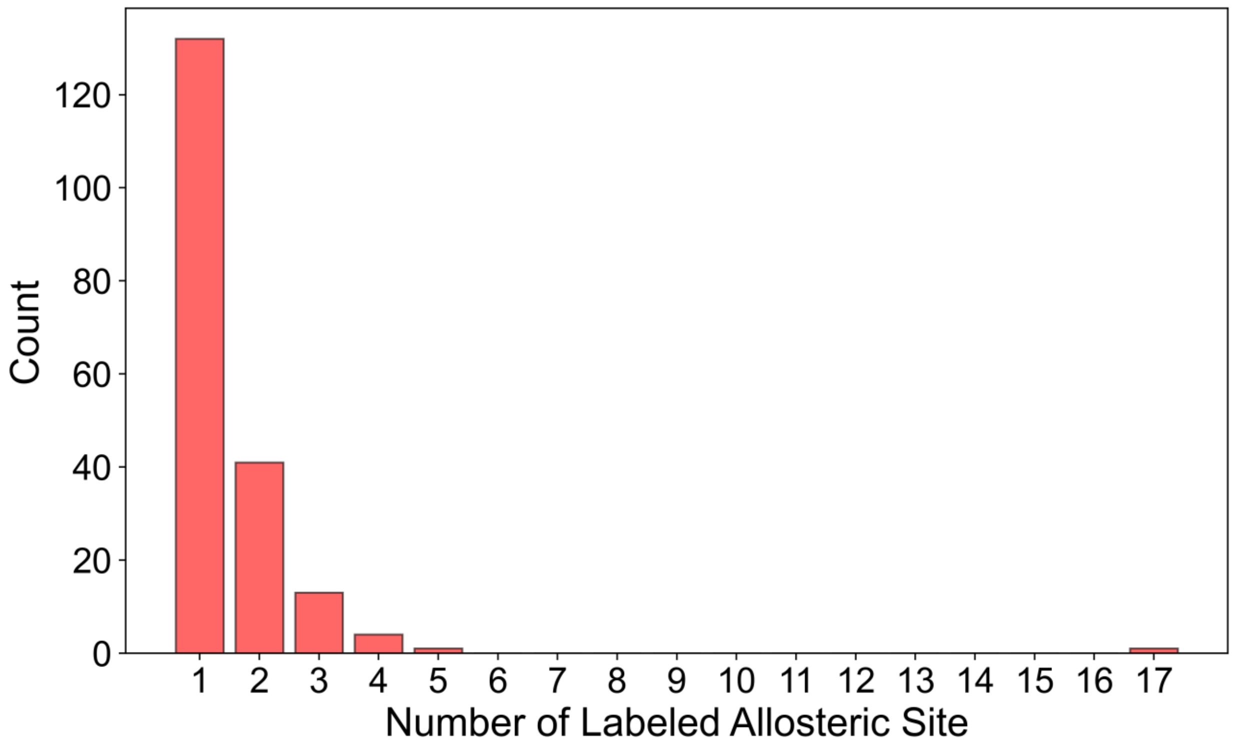

Figure S2. Labeling of detected pockets in 192 proteins. 291 of 3357 pockets are labeled as allosteric sites.

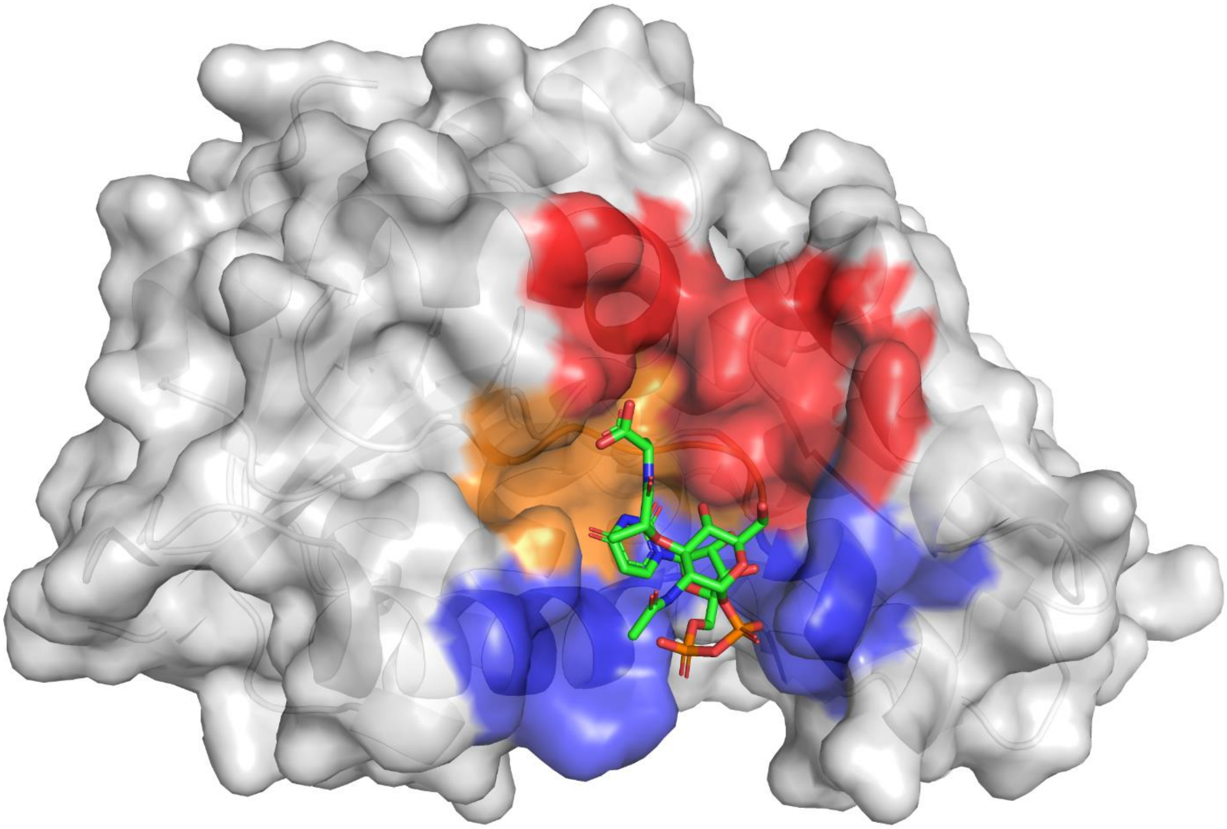

Figure S3. When using FPocket to detect pockets on glutamate racemase (PDB: 2JFZ), it splits the allosteric site binding uridine-5'-diphosphate-n-acetylmuramoyl-l-alanine (green) into 3 sub-pockets (red, orange and blue).

A

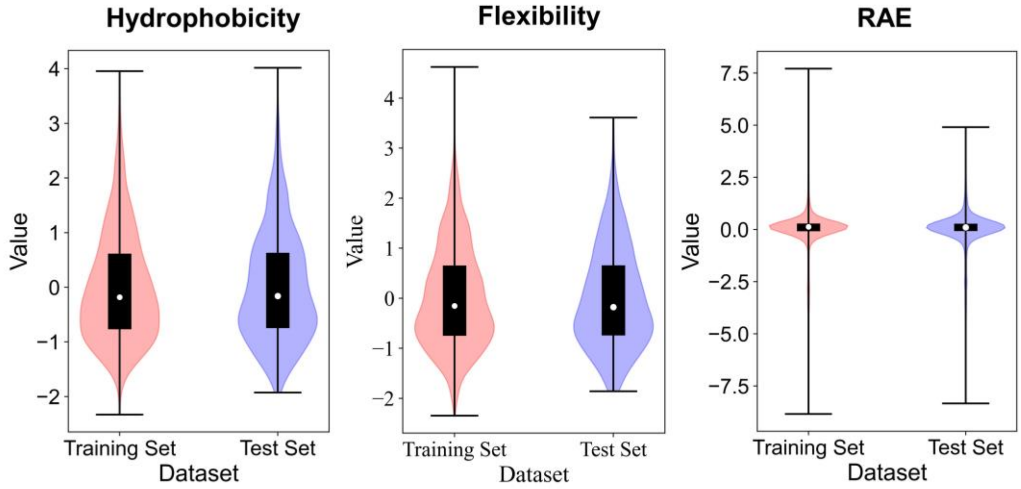

Mann-Whitney-U Test (H0: Distribution of feature data is the same in training and test set.)

Statistics	1121439.0	1126843.0	1128003.0
p-Value	0.869814	0.959025	0.922254

B

	Allosteric Site	Other Site	Chi-Square Test (H0: Ratio of "allosteric site" is the same in training and test set.)
Training Set	208	2223	Statistics: 0.09368<br>p-Value: 0.759550
Test Set	83	843	

Figure S4. Statistic test of (**A**) feature distribution (Z-value) and (**B**) ratio of positive (allosteric site)/negative samples (other site) between training and dataset. Mann-Whitney-U test and Chi-square test were performed using scipy package. Tates' correction was applied in Chi-square test.

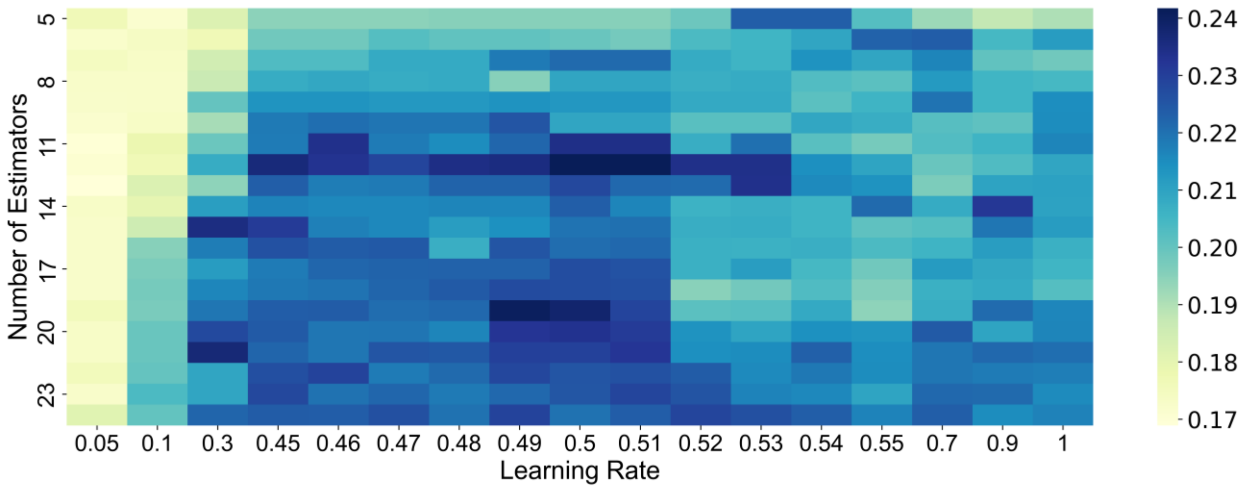

Figure S5. Grid search for super parameters in AdaBoost.

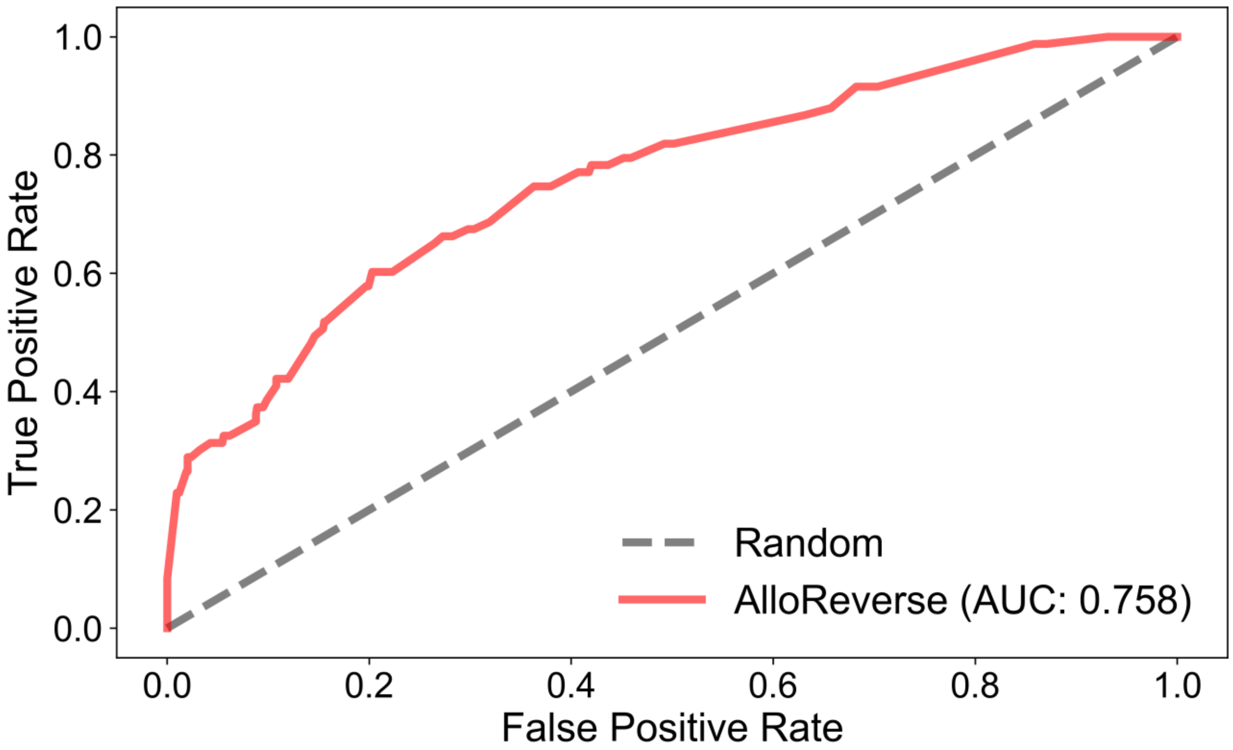

Figure S6. ROC curve of AlloReverse in predicting allosteric sites on test set.

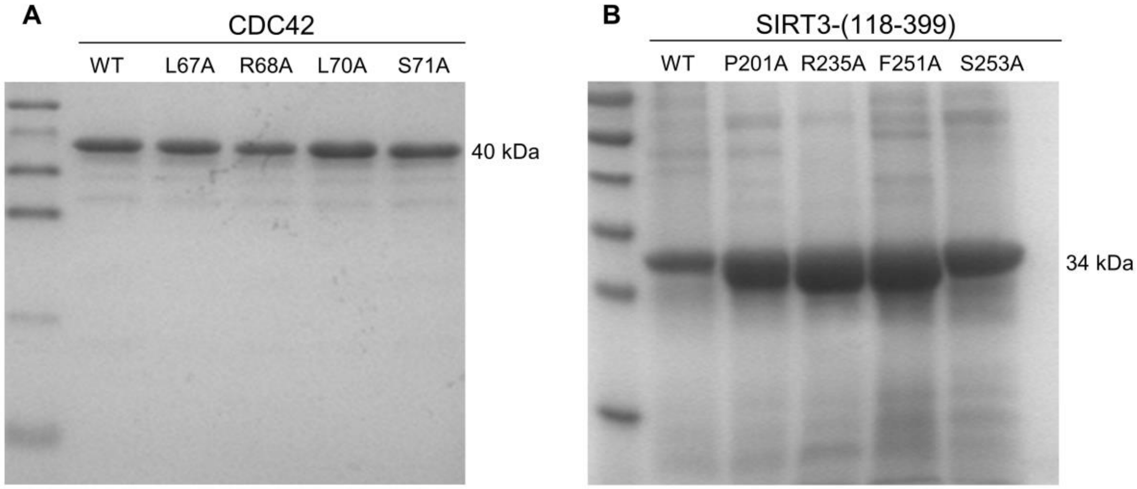

Figure S7. A coomassie-stained SDS-PAGE gel of purified (A) CDC42 (WT, L67A, R68A, L70A, and S71A) and (B) SIRT3-(118-399) (WT, P201A, R235A, F251A, and S253A).

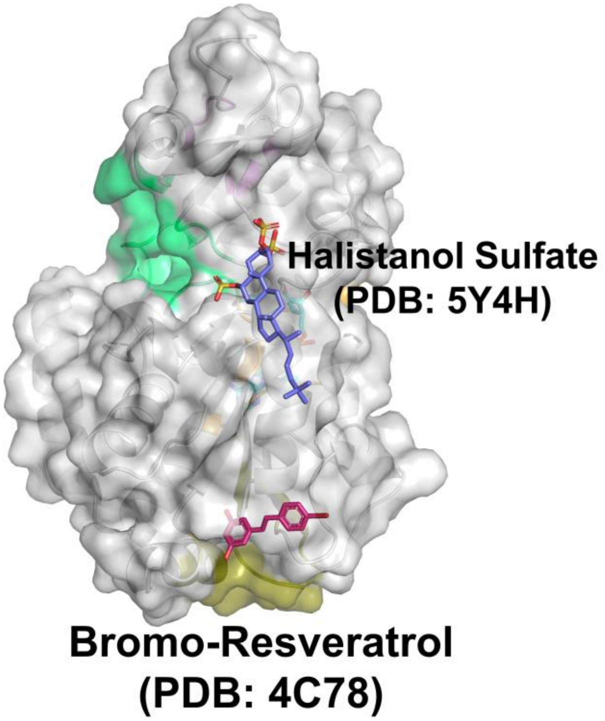

Figure S8. Comparison of detected allosteric sites and previously reported allosteric ligands on SIRT 3.

Section S4: Tables.

Table S1. Summary of training set.

Name of Protein	Uniprot ID	PDB ID (PDB:Chain(s))	Orthosteric Ligand (PDB:Chain:Residue Name: Residue ID)	Allosteric Ligand(s) (PDB:Chain:Residue Name: Residue ID)
Pyruvate kinase 1	P00549	1A3W:A	1A3W:A:PGA:1005	1A3W:A:FBP:1007
Ribose-phosphate pyrophosphokinase	P14193	1DKU:AB	1DKU:A:ABM:1003	1DKU:A:AP2:1002; 1DKU:B:AP2:1001
Fructose-1,6-bisphosphatase 1	P09467	1FBP:A	1FBP:A:F6P:336	2JJK:A:R15:1336; 1FRP:A:AMP:338; 1KZ8:A:PFE:738
Glucose-1-phosphate thymidylyltransferase	O27819	1G2V:A	1G2V:A:TTP:3500	1LVW:A:TYD:3002
Glucose-1-phosphate thymidylyltransferase 1	P37744	1H5R:A	1H5R:A:G1P:1294	1H5S:A:TMP:1292
Glutamate dehydrogenase 1, mitochondrial	P00366	1HWY:F	1HWY:F:NAD:507	1HWY:B:NAD:507; 1HWY:F:NAD:508
Sulfate adenylyltransferase	Q12650	1I2D:A	1I2D:A:ADX:574	1M8P:A:PPS:574
Uracil phosphoribosyltransferase	Q26998	1JLS:B	1JLS:B:PRP:301	1JLR:A:GTP:303
Insulin-like growth factor 1 receptor	P08069	1JQH:A	1JQH:A:ANP:301	3LW0:A:CCX:1
Phosphoenolpyruvate carboxylase	P00864	1JQN:A	1JQN:A:DCO:901	1FIY:A:ASP:884
Glucosamine-6-phosphate isomerase 1	P46926	1NE7:AB	1NE7:A:AGP:2298	1NE7:B:16G:2299
Kinesin-like protein KIF11	P52732	1Q0B:A	1Q0B:A:ADP:601	3ZCW:A:4A2:1366; 4BBG:A:V02:1370
Aspartate carbamoyltransferase regulatory chain	P0A7F3	1Q95:AG	1Q95:A:PAL:1001	4FYY:B:UTP:202; 4FYY:B:CTP:203; 4FYY:B:MG:204
2-dehydro-3-deoxyphosphoheptonate aldolase	Q9WYH8	4GRS:AC	1RZM:A:PEP:8002; 1RZM:A:E4P:8003	4GRS:A:TYR:401; 4GRS:A:TYR:402
Carbamoyl-phosphate synthase large chain	P00968	1T36:A	1T36:A:ADP:1088; 1T36:A:ADP:5007	1T36:A:ORN:1089; 1CE8:A:IMP:5012
Mitogen-activated protein kinase 8	P45983	1UKI:AB	1UKI:A:537:0	3O2M:A:46A:701
Response regulator PleD	B8GZM2	1W25:A	1W25:A:C2E:501	1W25:A:C2E:503; 1W25:A:C2E:505
Farnesyl pyrophosphate synthase	P14324	1YQ7:A	1YQ7:A:RIS:901	3N1V:F:3N1:1
Acetylglutamate kinase	Q9X2A4	2BTY:A	2BTY:A:NLG:1284	2BTY:A:ARG:1283
Serum albumin	P02768	2BXG:A	2BXG:A:IBP:2001	2BXA:A:C1F:2001; 3LU6:A:IMX:587
Threonine synthase 1, chloroplastic	Q9S7B5	2C2B:AB	2C2B:A:PLP:1163	2C2B:A:SAM:500; 2C2B:A:SAM:501; 2C2B:B:SAM:500; 2C2B:B:SAM:501
Aspartokinase 1, chloroplastic	Q9LYU8	2CDQ:A	2CDQ:A:LYS:600	2CDQ:A:SAM:1500
Tyrosine-protein phosphatase non-receptor type 5	P54829	2CJZ:A	2CJZ:A:PTR:1537	6H8S:A:FSZ:601

Serine/threonine-protein kinase Chk1	O14757	2E9N:A	2E9N:A:76A:1001	3F9N:A:38M:324
Glycogen phosphorylase, muscle form	P00489	2IEG:A	2IEG:A:PLR:903	2IEG:A:FRY:901
Focal adhesion kinase 1	Q05397	4EBW:A	2IJM:A:ATP:690	4EBW:A:0PF:700
Hepatitis C virus NS5B polymerase	Q99AU2	2IJN:A	2IJN:A:221:9366	2HAI:A:PFI:601
Lysine-sensitive aspartokinase 3	P08660	2J0X:AB	2J0W:A:ADP:820	2J0X:A:LYS:1451; 2J0X:B:LYS:1450
Myosin-2 heavy chain	P08799	2JHR:A	2JHR:A:AD9:1778	2JHR:A:PBQ:1780; 3BZ7:A:BL4:800
DNA-directed RNA polymerase subunit beta	Q8RQE9	2O5J:ABCDE	2O5J:D:APC:3999	2A69:C:RPT:8001
UDP-glucose pyrophosphorylase	Q4QDU3	2OEG:A	2OEG:A:UPG:5206	5NZM:A:9ET:601
S-adenosylmethionine synthase isoform type-2	P31153	5UGH:AC	2P02:A:SAM:2	5UGH:A:8AJ:401
KSHV protease	O36607	2PBK:A	2PBK:D:ACE:1; 2PBK:D:PRO:2; 2PBK:D:VAL:3; 2PBK:D:TYR:4; 2PBK:D:TBG:5; 2PBK:D:GLN:6; 2PBK:D:GG7:7	4P2T:A:24Q:201
Glutamine--fructose-6-phosphate aminotransferase	P53704	2PUV:B	2PUV:B:M6R:5004	2PUV:B:UD1:5003
Androgen receptor	P19091	2QPY:AB	2QPY:A:DHT:931	2QPY:A:4HY:1
Acetylglutamate kinase, chloroplastic	Q9SCL7	2RD5:A	2RD5:A:ADP:2000	2RD5:A:ARG:1000
Pyruvate kinase PKLR	P30613	2VGB:A	2VGB:A:PGA:581	2VGB:A:FBP:580
Glutamate racemase	Q836J0	2VVT:A	2VVT:A:DGL:1270	2VVT:A:I24:1269
Glucosamine-6-phosphate deaminase	P0A759	2WU1:A	2WU1:A:FGS:1268	2WU1:A:16G:1267
DNA gyrase subunit B	P66937	2XCS:BD	2XCS:F:RXV:1021	5NPK:B:94H:1502; 5NPK:D:94H:1503
Multifunctional 2-oxoglutarate metabolism enzyme	A0R2B1	2Y0P:A	2Y0P:A:TD7:2001	2Y0P:A:ACO:2228
C-C chemokine receptor type 7	P62576	6QZH:A	2YA7:A:ZMR:1776	6QZH:A:JLW:1502
Androgen receptor	P10275	2YHD:A	2YHD:A:TES:1920	2YHD:A:AV6:1921
Pyruvate kinase PKM	P14618	3BJT:B	3BJT:B:OXL:902	4B2D:A:SER:1532; 4G1N:A:NZT:603; 3H6O:A:FBP:541
Ribosomal protein S6 kinase alpha-3	P18654	3G51:A	3G51:A:ANP:480	5O1S:A:9HB:802
RTX toxin RtxA	Q9KS12	3GCD:A	3GCD:A:AZ0:213	3GCD:A:IHP:214
Glucokinase	P35557	3H1V:X	3H1V:X:GLC:500	3H1V:X:TK1:501
Isocitrate dehydrogenase [NADP] cytoplasmic	O75874	3INM:A	3INM:A:AKG:511	4UMX:A:VVS:1415; 6BKY:A:K32:502
Mitogen-activated protein kinase 14	Q16539	3KF7:A	3KF7:A:L9G:401	4E6C:A:0O8:500
Methionine--tRNA ligase	Q4QCD2	3KFL:A	3KFL:A:ME8:801	6SWX:A:LWN:801

Glutamate receptor 3	P19492	3LSX:A	3LSX:A:GLU:400	3LSX:A:PZI:802
Glutamate receptor 3	P19492	3M3F:A	3M3F:A:GLU:400	3M3F:A:P99:800
Pyruvate kinase PKM	P11974	3N25:A	3N25:A:PYR:1000	3N25:A:PRO:1200
Phospho-2-dehydro-3-deoxyheptonate aldolase AroG	O53512	3NV8:A	3NV8:A:PEP:467	3KGF:B:PHE:4188; 3KGF:B:PHE:9001; 3KGF:B:TRP:9004
Ribonucleotide reductase	O33839	3O0O:B	3O0O:B:GDP:1003	1XJF:B:DTP:1001
UDP-glucose 6-dehydrogenase	A0A0J9W ZA6	3PJG:A	3PJG:A:UGA:901	3PJG:A:UGA:902
Tyrosine-protein kinase ABL1	P00519	3PYY:A	3PYY:A:STI:3	3PYY:A:3YY:538
Poly [ADP-ribose] polymerase 14	Q460N5	5O2D:A	3Q6Z:A:APR:1	5O2D:A:9HH:1201
N-acetylglutamate kinase / N-acetylglutamate synthase	Q0ASS9	3S6H:A	3S6H:A:GLU:501	4KZT:A:ARG:501
Glutaminase kidney isoform, mitochondrial	D3Z7P3	4JKT:AD	3SS5:D:GLU:1	4JKT:D:04A:701
FLAVIVIRUS_NS2B/Peptidase S7	Q91H74	3U1I:CDE	3U1I:E:ARG:4; 3U1I:E:OAR:5	6MO0:A:JVJ:1201
Glutaminase kidney isoform, mitochondrial	O94925	3UO9:A	3UO9:A:GLU:600	3UO9:B:04A:2
FARNESYL PYROPHOSPHATE SYNTHASE	Q9HWY4	3ZOU:A	3ZOU:A:GPP:1297	3ZOU:A:6H6:1299
Acetylglutamate kinase	Q01217	3ZZH:C	3ZZH:C:NLG:1361	3ZZH:C:ARG:1362
Acetyltransferase Pat	O05581	4AVC:A	4AVC:A:ACO:1334	4AVC:A:CMP:400
Gag-Pol polyprotein	P12497	4BDZ:AB	4BDZ:A:19C:1382	4AHS:A:AKH:1216
Deoxynucleoside triphosphate triphosphohydrolase SAMHD1	Q9Y3Z3	4BZB:A	4BZB:A:DGT:700	4BZB:A:DGT:800; 4BZB:A:DGT:900
ANAEROBIC RIBONUCLEOSIDE-TRIPHOSPHATE REDUCTASE	Q9WYL6	4COJ:A	4COJ:A:CTP:653	4COJ:A:DTP:654; 4COJ:B:DTP:654
5'-AMP-activated protein kinase subunit gamma-1	P80385	4EAJ:C	4EAJ:C:ATP:401	4EAJ:C:AMP:403
RAC-alpha serine/threonine-protein kinase	P31749	4EKK:BD	4EKK:B:ANP:501	3O96:A:IQO:444
Glucose-1-phosphate thymidylyltransferase	Q9AGY4	4HO6:A	4HO6:A:UPG:300	4HO6:A:UTP:301
Caspase-6	P55212	4NBN:AB	4HVA:A:4HV:401	4NBN:B:2J7:401
Pyruvate kinase 1	P30615	4HYV:B	4HYV:B:PEP:1003	4HYW:A:FDP:503
Mitogen-activated protein kinase 7	Q13164	4IC7:A	4IC7:A:ANP:501	4ZSG:A:4QX:406
Citrate synthase	P0ABH7	4JAG:A	4JAG:A:OAA:501	4JAF:A:NAI:503
Aspartate carbamoyltransferase regulatory chain	N.A.	4KH0:AB	4KH0:A:PAL:401	4KH1:B:CTP:202; 4KH1:B:UTP:203; 4KH1:B:MG:204
Pyruvate kinase(Trypanosoma cruzi)	Q4D9Z4	4KS0:A	4KS0:A:MG:1001; 4KS0:A:OXL:1003	4KS0:A:FDP:1004
Muscarinic acetylcholine receptor M2	P08172	4MQS:A	4MQS:A:IXO:501	4MQT:A:2CU:502

Glutamate receptor 2	P19491	4N07:A	4N07:A:GLU:302	4N07:A:2J9:301
Nuclear receptor ROR-gamma	P51449	4NB6:A	4NB6:A:444:501	5C4T:A:4Y6:601
Putative cellulose synthase	Q3J125	4P00:A	4P00:A:UDP:918	4P00:A:C2E:919; 4P00:A:C2E:920
Deoxycytidylate deaminase	H6WFU3	4P9D:A	4P9D:A:TMP:204	4P9D:A:TTP:203
Cystathionine beta-synthase	P35520	4PCU:A	4PCU:A:PLP:602	4UUU:A:SAM:1546
ATP-dependent 6-phosphofructokinase	P00512	4PFK:A	4PFK:A:ADP:324	4PFK:A:ADP:326
Phospho-2-dehydro-3-deoxyheptonate aldolase	Q9K169	4UMB:A	4UMB:A:0V5:1353	4UC5:A:PHE:1354
ATP-dependent 6-phosphofructokinase, platelet type	Q01813	4XZ2:B	4XZ2:B:ADP:801	4XZ2:B:FBP:802
ATP phosphoribosyltransferase	Q5HSJ4	4YB5:ADE	4YB7:B:ATP:303	4YB5:A:HIS:304; 4YB5:D:HIS:302; 4YB5:E:HIS:303
3-phosphoinositide-dependent protein kinase 1	O15530	5ACK:A	5ACK:A:ATP:500	5ACK:A:SVQ:600
Neuronal acetylcholine receptor subunit alpha-7	P36544	5AFJ:ABCDE	5AFJ:A:L0B:1207	5AFJ:D:42R:1206; 5AFJ:E:42R:1206; 5AFJ:A:42R:1206; 5AFJ:B:42R:1206; 5AFJ:C:42R:1206; 5AFK:A:5VU:1207; 5AFK:C:5VU:1208; 5AFK:D:5VU:1206; 5AFK:E:5VU:1207; 5AFK:E:5VU:1208; 5AFN:E:OJD:1215; 5AFN:A:OJD:1215; 5AFN:B:OJD:1217; 5AFN:C:OJD:1215; 5AFN:D:OJD:1215
Inosine-5'-monophosphate dehydrogenase	Q9HXM5	4DQW:A	5AHN:A:IMP:1468	4DQW:A:ATP:501
Ribonucleoside-diphosphate reductase 1 subunit alpha	P00452	5CNS:A	5CNS:A:CDP:801	5CNS:A:DAT:802
Carbamoyl-phosphate synthase [ammonia], mitochondrial	P31327	5DOU:A	5DOU:A:ADP:2008; 5DOU:A:ADP:2009	5DOU:A:NLG:2010; 6UEL:A:Q5A:1602
Calcium-Sensing Receptor	N.A.	7DD5:A	5FBK:A:TCR:601	7DD5:A:YP1:1109; 7E6T:A:CA:903
Transitional endoplasmic reticulum ATPase	P55072	5FTJ:A	5FTJ:A:ADP:807	5FTJ:A:OJA:1001
Glutamate receptor 1	Q05586	5H8Q:AB	5H8Q:A:GLU:302	5H8Q:B:5YE:302
Chorismate Mutase	Q8NNL5	5HUD:AG	5HUD:G:TSA:111	5HUD:A:TRP:509
Pyruvate dehydrogenase kinase isozyme 2	Q15119	5J6A:A	5J6A:A:P46:501	2BU6:A:TF2:1386; 2BU7:A:TF3:1386; 2BU8:A:TF4:1379
Type II NADH:ubiquinone oxidoreductase	Q8I302	5JWC:A	5JWC:A:FAD:601	5JWC:A:4W0:609; 5JWC:A:4W0:610; 5JWC:A:4W0:611
k-ras gtpase	P01116	5KYK:A	5KYK:A:6ZD:201	4LUC:A:20G:203
Glutamate receptor ionotropic, kainate 1	P22756	5MFQ:AB	5MFQ:A:KAI:903	5MFQ:A:2J9:901; 5MFQ:A:2J9:902

Cell division protein FtsZ	P0A031	5MN5:B	6YD5:A:GDP:401	6YD5:A:OM8:404
Tyrosine-protein kinase ABL1	P00519	5MO4:A	5MO4:A:NIL:601	5MO4:A:AY7:602
Myosin-7	P12883	4PA0:A	5N6A:A:PHE:901	4PA0:A:2OW:1101; 5N69:A:2OW:904
FBP protein	O97193	5OFU:A	5OFU:A:F6P:402	5OFU:A:AMP:401
Aurora kinase A	O14965	5OS5:A	5OS5:A:ADP:401	5OS5:A:A8K:404
Aurora kinase A	O14965	5OSF:A	5OSF:A:ADP:401	5OSF:A:A9E:404
Humanized alpha-AChBP	N.A.	5OUG:A	5OUG:A:L0B:1101	5OUG:A:9Z0:1104; 5OUG:A:9Z0:1105
Tryptophan synthase beta chain	P9WFX9	5TCJ:AB	5TCJ:B:P1T:501	5TCJ:B:79V:505
HIV-1 reverse transcriptase	P03366	5UV5:A	5UV5:A:Y55:603	4KKO:A:1RE:601; 4KFB:A:1QP:601; 4IG3:A:J94:609
Phospho-2-dehydro-3-deoxyheptonate aldolase	Q9I000	5UXM:A	5UXM:A:PEP:501	5UXM:A:TRP:502
PopP2 protein	A0A0S4VB05	5W3X:A	5W3X:A:ACO:502	5W3X:A:IHP:501
Pyruvate kinase	P9WKE5	5WSB:A	5WSB:A:OXL:504	5WSB:A:AMP:501; 5WSB:A:G6P:502
Beta-2 adrenergic receptor	P07550	5X7D:A	5X7D:A:CAU:1206	5X7D:A:8VS:1205; 6N48:A:KBY:1403
Kynurenine 3-monooxygenase	Q84HF5	5Y66:A	5Y66:A:KYN:503	5Y66:A:7ZR:502
ATP phosphoribosyltransferase catalytic subunit	G7JFL4	6CZM:ABC	6CZM:A:AMP:401	6CZM:A:HIS:402; 6CZM:B:HIS:402; 6CZM:C:HIS:402
Glycogen phosphorylase, muscle form	P00489	6F3L:A	6F3L:A:PLP:901; 6F3L:A:CJW:902	3E3N:A:AMP:843; 3BCR:A:AZZ:940; 4MRA:A:QUE:901
Dual specificity mitogen-activated protein kinase kinase 7	O14733	6YZ4:A	6IB2:A:862:501	6YZ4:A:1E8:514
Guanosine 5'-monophosphate Reductase	Q57ZS7	6JIG:A	6JIG:A:5GP:601	6LK4:A:GTP:601
Protein arginine N-methyltransferase 5	O14744	6UXY:A	6K1S:A:CUX:700	6UXY:A:QKY:701
Cannabinoid receptor 1	P21554	6KQI:A	6KQI:A:9GF:1201	6KQI:A:9GL:1202
Arachidonate 5-lipoxygenase	P09917	6N2W:B	6N2W:B:30Z:702	6NCF:B:AF7:702
Deoxyhypusine synthase	P49366	6P4V:A	6P4V:A:NAD:501	6P4V:A:GC7:502
ATP phosphoribosyltransferase regulatory subunit	Q4FTX3	6R02:AG	6R02:G:PRP:301	6R02:A:HIS:401
IMP-specific 5'-nucleotidase, putative	A0A144A134	6RME:A	6RME:A:IMP:501	6RMD:D:ATP:501
Guanosine-3',5'-bis(Diphosphate) 3'-pyrophosphohydrolase	Q5SHL3	6S2U:A	6S2U:A:AMP:402	6S2U:A:GN3:403
L-lactate dehydrogenase A chain	P00338	6SBU:AB	6SBU:A:NAI:1002	6SBV:A:L5K:1001; 6SBV:B:L5K:1001
3-oxoacyl-[acyl-carrier-protein] reductase	V5VHN7	6T65:CD	6T62:A:NAP:301	6T65:C:MLH:301

Coenzyme A biosynthesis bifunctional protein	A0QWT2	6THC:B	6TGV:B:CTP:501	6THC:B:N9N:503
Gamma-aminobutyric acid receptor subunit beta-2	P47870	6X3Z:A	6X3Z:A:ABU:405	6X3X:A:DZP:406
SUMO-activating enzyme subunit 1	Q9UBE0	6CWY:D	6XOG:C:VAY:201	6CWY:D:FHJ:707
Galactokinase	P51570	6ZFH:A	6ZFH:A:QV2:402	6ZGX:D:S6V:401
High affinity nerve growth factor receptor	P04629	6D20:A	6D20:A:FQD:801	6D20:A:FQG:802
Replicase polyprotein 1ab	P0DTD1	7AXM:A	7AKU:A:RN2:401	7AGA:A:LZE:401; 7AXM:A:93J:502
Histone-lysine N-methyltransferase	Q9H7B4	7O2B:A	7O2B:A:UZQ:1001	6YUH:A:POW:503

Table S2. Summary of test set.

Name of Protein	Uniprot ID	PDB ID (PDB:Chain(s))	Orthosteric Ligand (PDB:Chain:Residue Name: Residue ID)	Allosteric Ligand(s) (PDB:Chain:Residue Name: Residue ID)
Seminal ribonuclease	P00669	11BG:A	11BG:A:U2G:130	11BG:A:U2G:131
Transforming protein	P61586	1A2B:A	1A2B:A:GSP:538	6KX3:A:8ZO:202
Cyclin-dependent kinase 2	P24941	1B38:A	1B38:A:ATP:381	3PXF:A:2AN:304; 3PXF:A:2AN:305; 6Q4D:A:HHT:302; 6Q4D:A:HHT:303
Hexokinase-1	P19367	1DGK:N	1DGK:N:ADP:922	1HKB:A:G6P:919
Glucose-1-phosphate thymidylyltransferase	Q9HU22	1G3L:A	1G3L:A:TRH:500	1G3L:A:TRH:501
Matrix metalloproteinase-9	P14780	5UE4:B	1GKD:A:STN:1448; 1GKD:A:BUM:1449	5UE4:B:5XQ:307
NAD-dependent malic enzyme, mitochondrial	P23368	1GZ3:A	1GZ3:A:ATP:601	1GZ3:A:FUM:605
Leukotriene A-4 hydrolase	P09960	1HS6:A	1HS6:A:BES:901	3FUD:A:692:710
Anthranilate synthase component 1	P00898	1I7Q:A	1I7Q:A:BEZ:1501	1I1Q:A:TRP:1001
Phospho-2-dehydro-3-deoxyheptonate aldolase, Phe-sensitive	P0AB91	1KFL:A	1KFL:A:PEP:1352	1OF6:A:DTY:1370
L-lactate dehydrogenase 2	E8ME30	1LTH:R	1LTH:R:NAD:321	1LTH:R:FBP:320
Ornithine decarboxylase	Q9TZZ6	1NJJ:A	1NJJ:A:ORX:602	1NJJ:A:GET:601
ATP-dependent 6-phosphofructokinase isozyme 1	P0A796	1PFK:A	1PFK:A:ADP:324	1PFK:A:ADP:326
Prothrombin	P00735	3PMA:CD	1SHH:B:0G6:301	3PMA:F:GU4:1; 3PMA:F:YYJ:2
NAD(P)-dependent glyceraldehyde-3-phosphate dehydrogenase	O57693	1UXV:A	1UXV:A:NAP:1502	1UXV:A:AMP:1503
Uracil phosphoribosyltransferase	Q980Q4	1XTU:A	1XTU:A:U5P:1250	1XTU:A:CTP:1260

Glycogen phosphorylase, muscle form	P11217	1Z8D:A	1Z8D:A:GLC:901	1Z8D:A:AMP:900; 1Z8D:A:ADE:902
Cathepsin K	P43235	5J94:A	1U9W:A:IHI:300	5J94:A:1XF:301
Uridylate kinase	P0A7E9	2V4Y:ABCDEF	2BNF:A:UTP:1242	2V4Y:A:GTP:1242; 2V4Y:B:GTP:1242; 2V4Y:C:GTP:1242; 2V4Y:D:GTP:1242; 2V4Y:E:GTP:1242; 2V4Y:F:GTP:1242
Glutamate racemase	P22634	2JFZ:A	2JFZ:A:DGL:1257	2JFZ:A:003:1256; 2JFN:A:UMA:1286
L-lactate dehydrogenase	P00344	2LDB:D	2LDB:D:NAD:13	2LDB:D:FBP:2
D-3-phosphoglycerate dehydrogenase	P0A9T0	2PA3:A	2PA3:A:NAI:450	2PA3:A:SER:451
Glycogen phosphorylase, liver form	P06737	2SKC:A	2SKC:A:GLC:998; 2SKC:A:PLP:999	3CEH:A:AVE:833; 1EM6:A:CP4:862; 3DD1:A:CFF:903
Androgen receptor	P10275	2YLO:A	2YLO:A:TES:1920	2YLO:A:YLO:1922
UDP-N-acetylglucosamine pyrophosphorylase, putative	Q386Q8	2YQS:A	2YQS:A:UD1:1001	4BQH:A:9VU:1539
Probable aspartokinase	Q57991	3C1N:AB	3C1M:A:ANP:472	3C1N:A:THR:471; 3C1N:B:THR:470
Amino-acid acetyltransferase	Q5FAK7	3D2P:A	3D2P:A:COA:437	3D2P:A:ARG:438
Isocitrate dehydrogenase kinase/phosphatase	Q8X607	3EPS:A	3EPS:A:ATP:1605	3EPS:A:AMP:1604
Pyruvate kinase	Q27686	3HQP:A	3HQP:A:ATP:1001	3HQP:A:FDP:700
4-hydroxy-tetrahydrodipicolinate synthase	P0A6L2	2ATS:AB	3I7S:A:PYR:298	2ATS:A:DLY:3003
HD domain protein	Q836G9	3IRH:AB	3IRH:B:DTP:459	3IRH:A:DGT:458; 3IRH:B:DGT:458; 4LRL:B:TTP:503
Tyrosine-protein kinase Abl1	P00520	3K5V:A	3K5V:A:STI:2	3K5V:A:STJ:1
Glutamate receptor 2	P19491	3LSF:B	3LSF:B:GLU:400	3LSF:B:PZI:802; 3LSF:E:PZI:802
Toxin B	Q189K3	3PA8:A	3PA8:A:621:300	3PA8:A:IHP:257
Serine/threonine-protein kinase PAK 1	Q13153	3Q4Z:A	3Q4Z:A:ANP:800	4ZLO:A:4PV:601
Glutamate receptor 2	P42262	3RNN:A	3RNN:A:GLU:281	3RNN:A:RNN:280
Ribonucleoside-diphosphate reductase large chain 1	P21524	5IM3:A	3S87:A:ADP:1002	3S87:A:DGT:1001; 5IM3:A:DTP:1002
Penicillin binding protein 2 prime	A0A0H3J PA5	3ZFZ:A	3ZFZ:A:AI8:1403	3ZFZ:A:1W8:1669; 3ZFZ:A:MUR:1670
GTPase HRas	P01112	4DLR:A	4DLR:A:GNP:208	4DLR:A:DTU:203
Isocitrate dehydrogenase [NADP], mitochondrial	P48735	4JA8:AB	4JA8:A:NDP:501	4JA8:A:1K9:502
U5 small nuclear ribonucleoprotein 200 kDa helicase	O75643	4KIT:B	4KIT:B:ADP:2201	5URJ:A:8LS:2202

Ribonucleoside-diphosphate reductase large subunit	P23921	4R1R:ADP	4R1R:A:GDP:763	3HNC:A:TTP:802; 3R1R:A:ATP:762
TDP-3-aminoquinovose-N-formyltransferase	F8RC03	4XCZ:A	4XCZ:A:FON:401	4XCZ:A:T3Q:403
P2Y purinoceptor 1	P47900	4XNW:A	4XNW:A:2ID:1101	4XNV:A:BUR:1101
Inosine-5'-monophosphate dehydrogenase	Q756Z6	5TC3:A	4XTI:A:IMP:601	5TC3:A:ATP:601; 5TC3:A:GDP:602; 5TC3:A:GDP:603
Bifunctional UDP-N-acetylglucosamine 2-epimerase/N-acetylmannosamine kinase	Q9Y223	4ZHT:A	4ZHT:A:UDP:501	4ZHT:A:NCC:502
GDP-mannose 4,6 dehydratase	O60547	5IN4:A	5IN4:A:GDP:1002	5IN4:A:6CK:1003
Fructose-1,6-bisphosphatase isozyme 2	O00757	3IFA:A	5K56:A:FBP:401	3IFA:A:AMP:339
Inorganic pyrophosphatase	P9WI55	5KDF:A	5KDF:A:POP:205	5KDF:A:6RU:201
Glucose-1-phosphate adenylyltransferase	P0A6V1	5L6V:A	5L6V:Q:GLC:1; 5L6V:Q:FRU:2	5L6V:A:AMP:501; 5W5R:A:PYR:503
Myosin-11	P10587	5M05:A	5M05:A:ADP:903	5M05:A:52E:901
CC chemokine receptor type 2 isoform B	P41597	5T1A:A	5T1A:A:73R:1201	5T1A:A:VT5:1202
Chimera of G-protein coupled receptor 52 and Flavodoxin	Q9Y2T5	6LI1:A	6LI1:A:FMN:1202	6LI0:A:EN6:1401
2-succinyl-5-enolpyruvyl-6-hydroxy-3-cyclohexene-1-carboxylate synthase	P9WK11	6O0J:C	6O0J:C:TPP:604	6O0J:C:DNA:601
Histone acetyltransferase p300	Q09472	6PGU:A	6PGU:A:COA:1701	6PGU:A:OK7:1702
G-protein coupled bile acid receptor 1	Q8TDU6	7CFN:R	7CFN:R:FX0:403	7CFN:R:FX0:401
D(1A) dopamine receptor	P21728	7LJC:R	7LJC:R:SK0:501	7LJC:R:G4C:502
4-hydroxy-tetrahydrodipicolinate synthase 1, chloroplastic	Q9LZX6	7MDS:A	7MDS:A:YXP:501	6VVH:A:LYS:401
